# Whole genome sequence analysis of ESBL-producing *Escherichia coli* recovered from New Zealand freshwater sites

**DOI:** 10.1099/mgen.0.000893

**Published:** 2022-10-06

**Authors:** Sara A. Burgess, Marie Moinet, Gale Brightwell, Adrian L. Cookson

**Affiliations:** ^1^​ mEpiLab, School of Veterinary Science, Massey University, Palmerston North 4410, New Zealand; ^2^​ AgResearch Ltd, Hopkirk Research Institute, Massey University, Palmerston North 4410, New Zealand; ^3^​ New Zealand Food Safety Science and Research Centre, Massey University, Palmerston North, New Zealand

**Keywords:** antimicrobial resistance, ESBL, *Escherichia coli*, freshwater

## Abstract

Extended-spectrum beta lactamase (ESBL)-producing *Escherichia coli* are often isolated from humans with urinary tract infections and may display a multidrug-resistant phenotype. These pathogens represent a target for a One Health surveillance approach to investigate transmission between humans, animals and the environment. This study examines the multidrug-resistant phenotype and whole genome sequence data of four ESBL-producing *E *. *coli* isolated from freshwater in New Zealand. All four isolates were obtained from a catchment with a mixed urban and pastoral farming land-use. Three isolates were sequence type (ST) 131 (CTX-M-27-positive) and the other ST69 (CTX-M-15-positive); a phylogenetic comparison with other locally isolated strains demonstrated a close relationship with New Zealand clinical isolates. Genes associated with resistance to antifolates, tetracyclines, aminoglycosides and macrolides were identified in all four isolates, together with fluoroquinolone resistance in two isolates. The ST69 isolate harboured the *bla*_CTX-M-15_ gene on a IncHI2A plasmid, and two of the three ST131 isolates harboured the *bla*_CTX-M-27_ genes on IncF plasmids. The last ST131 isolate harboured *bla*_CTX-M-27_ on the chromosome in a unique site between *gsp*C and *gsp*D. These data highlight a probable human origin of the isolates with subsequent transmission from urban centres through wastewater to the wider environment.

## Data Summary

All genome data for this study have been deposited in GenBank. Illumina sequence reads and Unicycler assemblies were deposited under accession PRJNA837629. The New Zealand ST131 and ST69 isolates with their accession details are given in Table S1 (available in the online version of this article). The supporting external data included in this study can be found under Bioprojects PRJDB4303, PRJNA531554, PRJNA600954, PRJNA398288 and PRJNA576546. The authors confirm all supporting data and protocols have been provided within the article or through supplementary data files.

**Impact Statement**

We describe the phenotypic and genetic basis for multidrug-resistant *Escherichia coli* isolated from freshwater in New Zealand. Comparative phylogenetic analysis with clinical isolates provides evidence of a human source and transmission from wastewater to the wider environment, highlighting the need for a One Health approach to investigate antibiotic-resistant bacterial transmission.

## Introduction

Antimicrobial resistance (AMR) is becoming an increasing problem in the treatment of community-acquired infections [1, 2]. In New Zealand, extended spectrum beta lactamase (ESBL)-producing *Escherichia coli* are commonly associated with multidrug-resistant urinary tract infections (UTIs) [3].

A key step in reducing the dissemination of AMR is through understanding where transmission occurs. An important pathway for the community spread of antimicrobially resistant bacteria is through person-to-person transmission [4], but other transmission pathways may also be relevant, including contact with animals, ingestion of food products or indirectly through contaminated waterways [5–8]. Recreational swimming has been identified as a risk factor for ESBL-producing *E. coli *-associated community infections [9]. Waterways including rivers, lakes and some beach waters have been identified as a vector for ESBL-producing *E. coli* [10–16], with the main origin of these bacteria being human faeces rather than emergence of AMR within the waterway itself [17]. It has been found that the concentration of ESBL-producing *E. coli* can be high (1×10^2^–1×10^5^ c.f.u. l^–1^) in treated sewage being discharged into waterways [16]. Genetically similar isolates have also been identified in the few studies that have been undertaken comparing water with clinical ESBL-producing isolates [10, 18].

In New Zealand, *E. coli* levels in waterways are measured and faecal source tracking has identified the main sources of faecal pollution in these waterways [19, 20]. However, little is known about the source of antimicrobially resistant *E. coli* bacteria. Here we characterize four ESBL-producing *E. coli* isolated from a New Zealand stream and explore their genetic relationship with New Zealand human clinical isolates.

## Methods

### Sample sites and collection

**Fig. 1.**
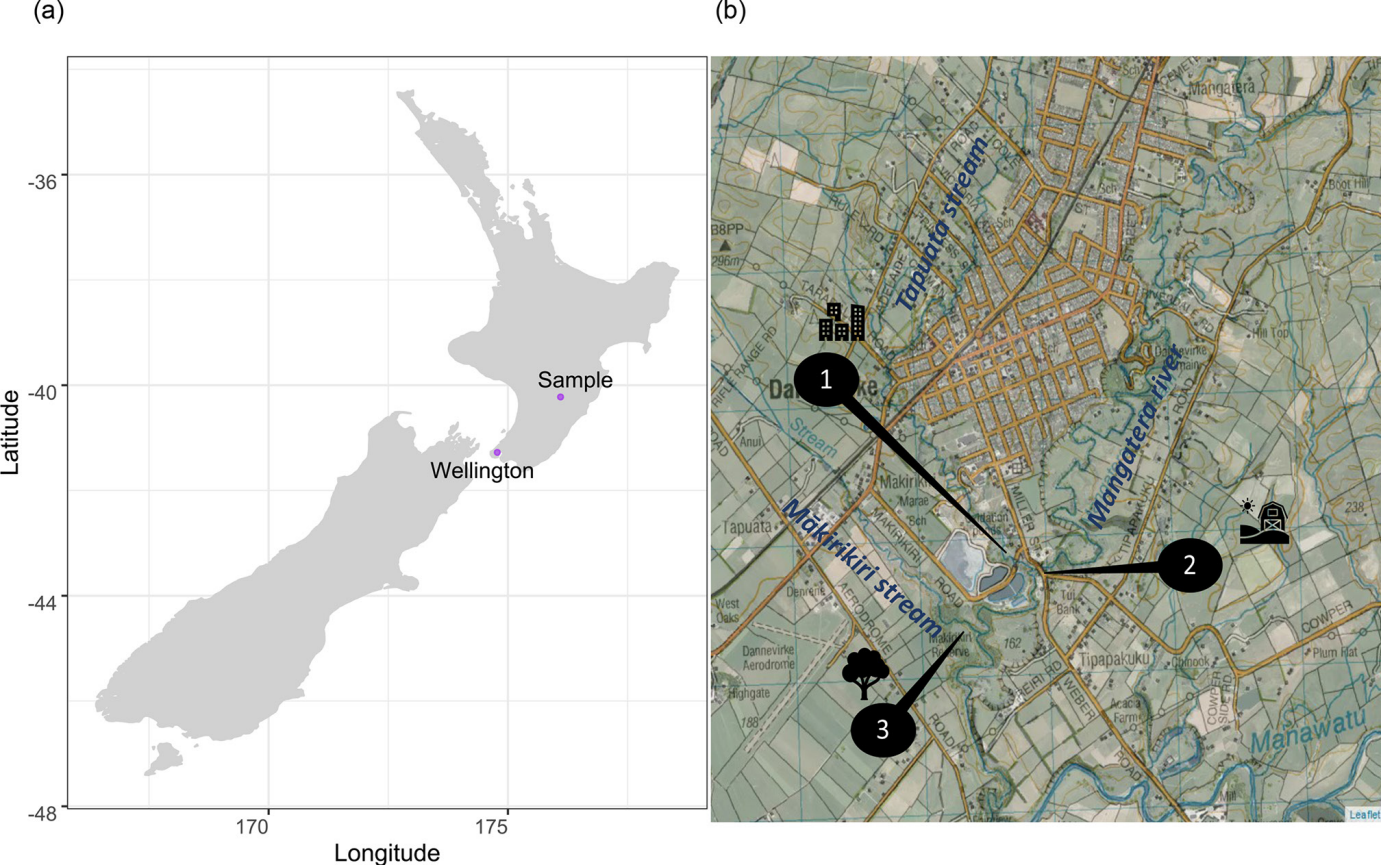
Sample site location. (**a**) New Zealand map showing the sample site location and (**b**) a topographical map illustrating the three sample sites.

Three freshwater sites with separate catchments were visited on 11 occasions over a 13 month period from March 2020 to March 2021. All three sites are in the general vicinity of Dannevirke in the Tararua region of the lower North Island, New Zealand (Fig. 1). Site 1 is on the Tapuata Stream (40° 13′ 25.32″ S 176° 05′ 58.91″ E) with a mixed urban and pastoral (ruminant livestock) farming land-use; Site 2 is on the Mangaterā River (40° 13′ 28.08″ S 176° 06′ 05.67″) with a pastoral (ruminant livestock farming) land-use; and Site 3 is on the Mākirikiri Stream (40° 13′ 38.01″ S 176° 05′ 39.35″ E) also with a pastoral (ruminant livestock) farming land-use. The Tapuata Stream sample site (Site 1) is approximately 200 m from the confluence with the Mangaterā Stream; the Mangaterā Stream sample site (Site 2) is approximately 20 m upstream of this confluence. The Mākirikiri Stream (Site 3) is approximately 250 m from its confluence with the Mangaterā Stream, and 600 m downstream of the Mangaterā Stream confluence (Fig. 1b).

On each sampling occasion, water was stored in a chilly bin with frozen ice blocks at approximately 4 °C and processed within 4 h at the Hopkirk Research Institute (Palmerston North).

### Bacterial culture and antibiotic susceptibility testing

Water (500 ml) was filtered through a 0.45 µm nitrocellulose filter using a bench-top negative pressure system, and incubated in 10 ml EC broth (Fort Richard) for 18 h at 35 °C. After enrichment, 10 µl of broth was inoculated onto CHROMagar ESBL (Fort Richard), and streaked for individual colonies. Agar plates were incubated for 18 h at 35 °C and examined for pink colonies indicative of ESBL-producing *E. coli *. In parallel with the recovery of ESBL-producing *E. coli *, 500 ml of water was filtered through a separate 0.45 µm filter, and incubated in 20 ml Bolton’s broth (Fort Richard) for enrichment of *Campylobacter* species as part of a parallel study. The broth was incubated at 42 °C in a microaerobic atmosphere (85 % N_2_, 10 % CO_2_, 5 % O_2_) for 48 h before an aliquot was plated onto modified charcoal-cefoperazone-deoxycholate agar (mCCDA) agar (Fort Richard) with a sterile cotton swab. mCCDA plates were examined after 48 h of incubation at 42 °C in a microaerobic atmosphere.

Putative ESBL-producing *E. coli* strains were sub-cultured onto CHROMagar ECC and MacConkey agar plates, then purified on sheep blood agar plates and identified using MALDI-TOF MS using the full ‘in tube formic acid extraction’ method (Bruker) [21]. Primary evaluation for AMR was undertaken against cefotaxime (30 µg), cefoxitin (30 µg), ceftazidime (30 µg) or cefpodoxime (10 µg), tetracycline (30 µg), streptomycin (10 µg) and ciprofloxacin (5 µg), and interpreted according to CLSI guidelines using Kirby–Bauer disc diffusion tests [22]. The AmpC and ESBL AMR phenotype was confirmed for isolates resistant to either cefoxitin and cefotaxime and/or cefpodoxime according to EUCAST guidelines using either a three-disc (D69C AmpC disc test; Mast Group) or double-disc comparison assay (D62C cefotaxime and D64C ceftazidime ESBL disc tests; Mast Group), respectively [23]. The AmpC-producing *E. coli* NZRM4402 and the ESBL-producing *Klebsiella pneumoniae* NZRM3681 were used as positive controls in the AmpC and ESBL confirmatory disc assays, respectively, and the susceptible *E. coli* NZRM916 was used as a negative control.

### Whole genome sequencing and assembly

Genomic DNA was extracted from the ESBL-producing isolates grown in lysogeny broth, using the Promega wizard genomic DNA purification kit as previously described [24]. Sequencing was performed using both short- and long-read technologies. For short-read sequencing, libraries were prepared using the Nextera XT DNA library preparation kit (Illumina) and sequencing was performed using an Illumina HiSeq X with 2×125 bp paired-end reads (Novagene). Raw sequence reads were trimmed and assembled using the Nullarbor bioinformatics pipeline (v.2.0.20181010) with default settings [25]. The trimming of reads in this pipeline was carried out using trimmomatic (v.0.39) [26]. Long-read sequencing using Oxford Nanopore Technologies (ONT) was then carried out as previously described [24] followed by base-calling using Guppy (v.5.0.7). The ONT reads were demultiplexed using qcat (v.1.1.0), trimmed using porechop (v.0.2.4) and filtered using Filtlong (v.0.2.0) in which 95 % of the reads were kept with a minimum length of 1000 bp and target number of bases of 500 Mb (100× depth). A hybrid assembly using both the illumina and ONT reads was carried out using unicycler (v.0.4.9b).

### Genome sequences and analyses

The genome sequences of the ESBL-producing *E. coli* isolated from the freshwater sites were compared with the genome sequences of 271 other New Zealand sequence type (ST)131 and ST69 strains previously sequenced by other institutes (Table S1) [3, 19, 27, 28]. The sequence reads were downloaded from the NCBI Sequence Read Archive database and processed using the Nullarbor pipeline (v.2.0.20191013). As part of this pipeline, the reads were re-assembled using SKESA (v. 2.4.0) and re-annotated using Prokka (v.1.14.6) [29, 30]. Additionally, resistance and virulence genes were identified with ABRicate (v.1.0.1) using the NCBI AMRFinderplus (v. 2021-03-28) and VirulenceFinder (v. 2021-03-28) databases respectively [31–33]. SNP analyses were undertaken using Snippy (v. 4.6.0) [34], using hybrid assemblies for strains AGR6128 and AGR6137 as references for the ST131 and ST69 SNP analyses, respectively. The SNP alignments were used to generate a maximum-likelihood tree, using a general time-reversible model with the Randomised Axelerated Maximum Likelihood (RAxML) Next-Generation tool [35]. The resulting tree was visualized with the Interactive Tree of Life (iTOL v. 6.5.8) [36].

### Plasmid analysis

The closest plasmid relatives (Table S2) [37–45] were determined using the plasmid database, PLSDB (v.2.1.1), with default settings and the fasta file was downloaded and re-annoated using Prokka (v.1.13.3). The program Roary (v.3.8.2) [46], using the Prokka-generated GFF file as input, was used to generate a core-genome nucleotide sequence alignment with PRANK of the IncF plasmids. A Neighbor-Net was constructed in Splits Tree (v.4.14.8) [47], using the core-genome alignment. Plasmid and resistance cassette comparisons were carried out in EasyFig (v.2.2.2) [48]. Plasmid incompatibility and sequence types were determined using pMLST (v. 2.0) and PlasmidFinder (v. 2.0) [49].

## Results and discussion

### Summary of sample sites

Three separate freshwater sites were each sampled on 11 occasions over a 13 month period. No ESBL-producing *E. coli* were identified from Site 2 or Site 3. However, four ESBL-producing *E. coli* (AGR4587, AGR5151, AGR6128 and AGR6137) were isolated from Site 1, Tapuata Stream (Dannevirke, New Zealand), and all four strains were isolated using mCCDA agar plates. Both Bolton’s broth and mCCDA agar have previously been problematic for isolating *Campylobacter,* with the increasing isolation of ESBL-producing *Enterobacteriaceae* [50, 51]. No ESBL-producing *E. coli* were isolated using enrichment in EC broth followed by plating onto CHROMagar ESBL, although subsequent subculture confirmed that the four ESBL-producing *E. coli* were able to grow on CHROMagar ESBL. This highlights the difficulties with using various culture-based methods for determining the prevalence of ESBL-producing *E. coli* in different sources [52].

Although much of the Tapuata Stream catchment is situated to the north-west of Dannevirke, a small tributary, the Mangapurupuru Stream, runs through and beneath the town of Dannevirke, flowing into the Tapuata Stream about 200 m upstream of Site 1. Studies have found ESBL-producing *E. coli* and other antibiotic-resistant bacteria in waterways where treated sewage outlets are located [53]. Although our sampling site was not located downstream of a treated sewage outlet, there may have been leakage from ageing wastewater infrastructure or raw sewage overflow. During high rainfall events raw sewage may overflow into storm water drains. However, in this study all the ESBL-producing *E. coli* were isolated during the summer or early autumn when rainfall was low. Previous studies have found a higher prevalence of third-generation cephalosporin-resistant *E. coli* in autumn [18, 54].

### Genetic relatedness

Whole genome sequence analysis determined that three of the four ESBL-producing *E. coli* strains were ST131 and one strain was ST69 (Table 1), with the difference in the number of SNPs ranging from 92 to 1066 between the three ST131 strains (Table S3). ST131 has previously been shown to be the predominant sequence type (41–54 %) amongst New Zealand clinical *E. coli* isolates, and ST69 has a prevalence of approximately 3 % [3, 55].

**Table 1.** Summary of the four ESBL-producing *E. coli* isolated from the Tapuata Stream

Isolate	Collection date	Phylo-group	Sequence type	Serotype	fimH type	Phenotype	Genome size (bp)	Accessions	No. of contigs	GC content (%)	AMR phenotype*
AGR4587	2 Mar 2020	B2	ST131	O16:H5	*fimH41*	ESBL	5 226 416	CP097360–CP097362	3	50.59	ESBL, STR, TET
AGR5151	1 Oct 2020	B2	ST131	O16:H5	*fimH42*	ESBL	5 027 765	CP097370–CP097372	3	50.71	ESBL, STR, TET
AGR6128	23 Feb 2021	B2	ST131	O16:H5	*fimH41*	ESBL	4 998 509	CP097367–CP097369	3	50.76	ESBL, STR, TET, CIP
AGR6137	9 Mar 2021	D	ST69	O15:H6	*fimH27*	ESBL	5 426 718	CP097363–CP097366	4	50.54	ESBL, STR, TET, CIP

*ESBL: extended-spectrum beta-lactamase; STR: streptomycin; TET: tetracycline; CIP, ciprofloxacin.

**Fig. 2.**
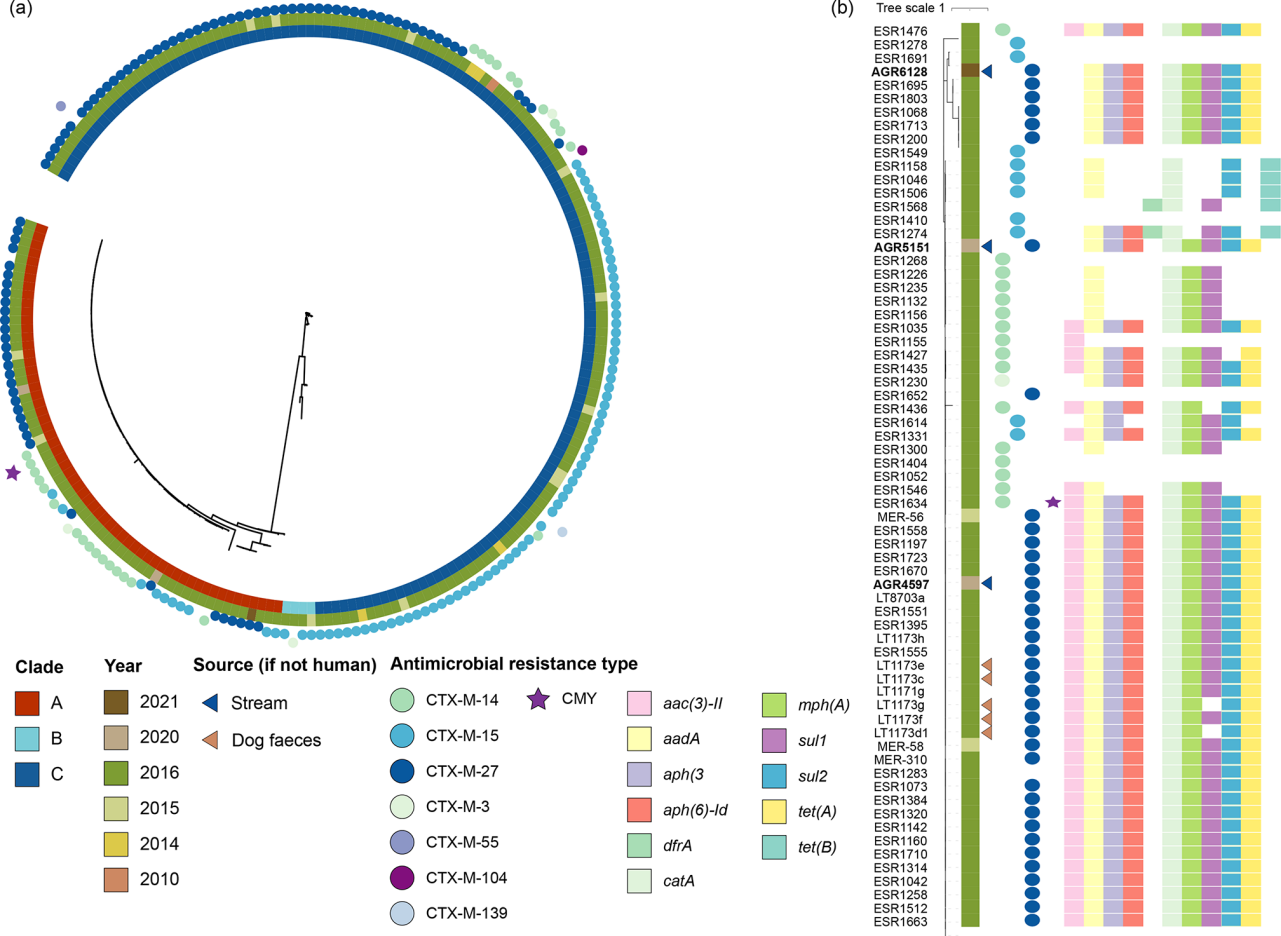
Core SNP comparison of the ST131 isolates from humans, dogs and water. Maximum-likelihood trees of New Zealand ST131 isolates (*n*=245), reconstructed using 14 296 core SNPs (**a**) and clade A ST131 isolates (*n*=66), reconstructed using 4 132 core SNPs (**b**). The genomes sequenced in this study are indicated in bold.

**Fig. 3.**
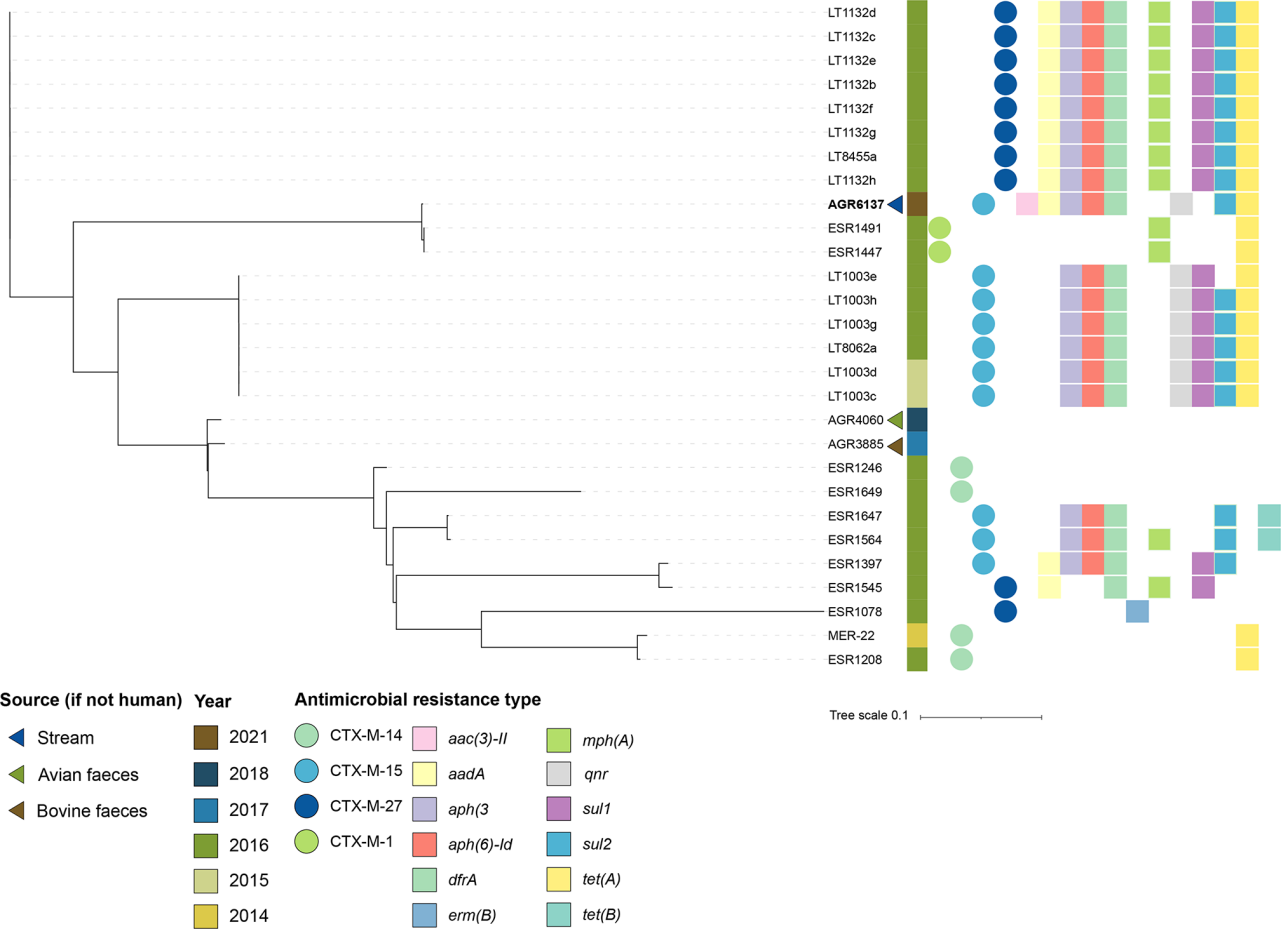
Core SNP comparison of the ST69 isolates from humans, animals (bovine and avian) and water. Maximum-likelihood trees of New Zealand ST69 isolates (*n*=27) reconstructed using 15 812 SNPs. The genome sequenced in this study is indicated in bold.

A phylogenetic comparison with other previously sequenced New Zealand ST131 and ST69 strains demonstrated that the four water strains were closely related to New Zealand clinical strains (Figs 2 and 3). ST131 is the predominant lineage associated with ESBL-associated human urinary tract and blood infections globally [56, 57], although other lineages such as ST69 are on the rise [56, 58]. Both ST131 and ST69 strains have been isolated from other animals, particularly poultry and dogs, worldwide [27, 59–61].

The three ST131 strains belonged to clade A and all harboured a *bla*_CTX-M-27_ gene. Two (AGR4587 and AGR6128) of the three ST131 water strains had a *fimH41*-type allele whereas AGR5151 had a *fimH42* allele. ST131 clade A strains frequently carry a *fimH41* allele [40, 62]. The close clustering (<10 SNPs) of these three water strains with clinical strains previously isolated in New Zealand suggests that the origin of these strains is humans. Additionally, there was evidence of clonal spread of CTX-M-27-producing *E. coli* within New Zealand over at least the past 6 years. The ST69 strain AGR6387 harboured the *bla*_CTX-M-15_ gene and clustered (43–44 SNPs) with two ESBL-producing (CTX-M-1 variant) clinical strains isolated from humans in 2016. In previous studies, CTX-M-15 has been the dominant variant identified in ESBL-producing *E. coli* isolated from surface waters in Europe [10, 18, 63].

### Distribution of AMR and virulence genes

The four water strains all displayed an ESBL-producing phenotype in agreement with their genotype. A multidrug resistance phenotype and genotype was also observed in the four water isolates (Table 1, Fig. 2b,c) with the genes associated with resistance comprising the four antibiotic classes: beta-lactams, antifolates (*dfrA17*, *sul1* and *sul2*), tetracyclines [*tet(A*)), aminoglycosides (*aac(3)-IId, aadA5, aph(6)-Id, aph(3″)-Ib*), and macrolides [*mph(A*)]. Additionally, the ST131 strain AGR6128 harboured mutations in both the *gyrA* and *parC* genes, in concordance with its ﬂuoroquinolone-resistance phenotype (Table 2). The acquisition of fluoroquinolone resistance in clade A ST131 strains is reported to be rare, with fluoroquinolone resistance being more commonly associated with the C2 H30 subclade [64]. However, a recent study found ST131 clade A strains isolated from wastewater harboured a higher prevalence of fluoroquinolone resistance compared with clade C strains [65]. In our study, the ST69 strain AGR6137 carried a *qnrB1* gene, which has previously been found to be carried by *bla*_CTX-M-15_ plasmids [66–68] but to our knowledge rarely in *bla*_CTX-M-27_ plasmids.

**Table 2.** Amino acid substitutions in GyrA, ParC and ParE of ESBL-producing *E. coli* isolated from the Tapuata Stream

	GyrA	ParC	ParE	Ciprofloxacin resistance phenotype
AGR4587	S83L		I529L	I
AGR5151	S83L		I529L	I
AGR6128	S83L, D87N	S80I	E460D	R
AGR6137	None	None	None	R

A multidrug resistance genotype (ranging from three to ten genes) was observed in most of the New Zealand ESBL-producing strains isolated from humans: 55 of the 64 (86 %) clade A ST131 strains and 20 of the 25 (80 %) ST69 strains. Multidrug-resistant ESBL-producing *E. coli* have been isolated from waterways worldwide [66, 69].

All four strains carried multiple virulence genes associated with extraintestinal pathogenicity [70, 71], such as *papA, papC, sfaC, afaC, kpsM* and *iutA* (Table 3). In addition, they carried the toxin genes *sat* (carried by strains AGR4587, AGR5151 and AGR6128) and *vat* (carried by AGR6137) as well as the siderophore gene *chuA* (carried by strains AGR4587, AGR5151 and AGR6128), which are genes typical of uropathogenic *E. coli *. This supports the notion that that these water strains originated from humans. No Shiga-toxin-associated genes were detected.

**Table 3.** Virulence genes detected among the four ESBL-producing *E. coli* isolates

	Virulence genes	AGR4587	AGR5151	AGR6128	AGR6137
Adhesin	*afaA*	−	+	−	−
	*clpV/tssH*	−	−	−	+
	*csg operon*	+	+	+	+
	*draP*	−	+	−	−
	*fim operon*	+	+	+	+
	*Hcp/tssD*	−	−	−	+
	*papA*	+	+	+	−
	*papC*	+	−	−	−
	*papEFGH*	+	−	−	−
	*sfa*	−	−	−	+
Toxin	*hlyABCD*	+	−	−	−
	*cnf*	+	−	−	−
	*pic*	+	+	+	−
	*sat*	+	+	+	−
	*vat*	−	−	−	+
Siderophore	*chuA*	+	+	+	−
	*irp1 and irp2*	+	+	+	+
	*iuc operon*	+	+	+	+
	*iutA*	+	+	+	+
	*iroN*	−	−	−	+
Other	*kpsM*	+	+	+	−

### Mobile genetic elements analysis

The ST69 strain harboured an IncHI2A plasmid encoding a *bla*_CTX-M-15_ gene as well as an IncF plasmid (Table 4). The three ST131 strains harboured IncF plasmids (Table 4), with two strains encoding the *bla*_CTX-M-27_ gene on the plasmid. The third ST131 strain, AGR4587, harboured *bla*_CTX-M-27_ on the chromosome, which was flanked by the insertion sequence elements ISEcp1 (belonging to the IS1380 family of insertion sequences) and IS903B. The insertion site of the *bla*_CTX-M-27_ gene was between the *gspC* and *gspD* genes, a possible unique site that to our knowledge has not been previously described [72, 73]. ISEcp1 insertion sequence elements have been commonly associated with the *bla*_CTX-M_ genes [3, 74, 75], and studies suggest it was also involved with the original mobilization of the *bla*_CTX-M_ gene from the chromosome of *Kluyvera* species to a plasmid [76]. The *bla*_CTX-M_ genes have been reported to be chromosomally encoded (particularly the *bla*_CTX-M-15_ variant) [64, 72–74, 77], but to our knowledge this is the first report of a chromosomally encoded *bla*_CTX-M-27_. All five plasmids contained the genes required for conjugation, including *traD* (encoding a coupling protein) and *traI* (encoding a relaxome).

**Table 4.** Plasmid characteristics

Plasmid	Size (bp)	Plasmid type	pMLST*	No. of CDS*	Beta-lactamase genes	AMR genes
pAGR4587	145, 705	Col156/IncFIB/IncFII	[F29:A-:B10]	179	*bla*_TEM-1_	*aac(3)-IId, aadA5, aph(6)-Id, aph(3″)-Ib, dfrA17, mph(A), qacEdelta1, sul1, sul2, tetA*
pAGR5151	137, 735	Col156/IncFIB/IncFII	[F2:A-:B10]	166	*bla*_CTX-27_	*aadA5, aph(6)-Id, aph(3″)-Ib, dfrA17, mph(A), qacEdelta1, sul1, sul2, tetA*
pAGR6128	122, 671	IncFIA/ IncFII/ Col156/IncFIB	[F1:A2:B20]	142	*bla*_CTX-27_	*aadA5, aph(6)-Id, aph(3″)-Ib, dfrA17, mph(A), qacEdelta1, sul1, sul2, tetA*
pAGR6137a	269, 522	IncHI2A	1	301	*bla*_TEM-1,_ *bla*_OXA-1,_ *bla*_CTX-M-15_	*aac(3)-IId, aadA1, aph(6)-Id, aph(3″)-Ib, catA1, dfrA14, qnrB1, sul2, tetA*
pAGR6137b	158, 302	IncFIA/IncFIC/IncFII	[F18:A5:B58]	154	*bla*_TEM-1_	

*pMLST: plasmid multi-locus sequence type; CDS: coding sequence.

**Fig. 4.**
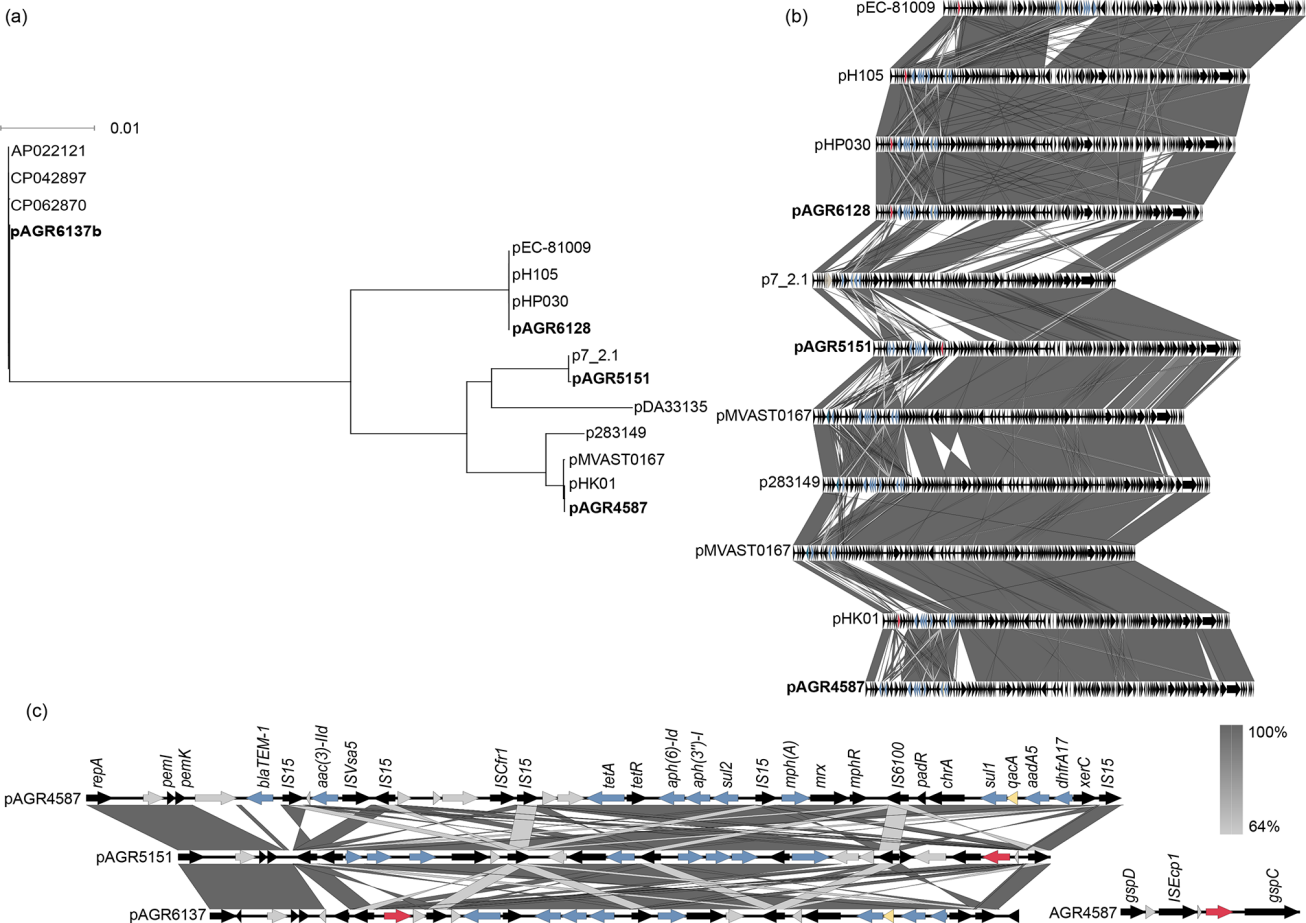
Comparison of the IncF plasmids with their closest relatives. A neighbour-joining tree was reconstructed using a core-genome alignment generated by Roary (v.3.8.2) (**a**). Genetic maps and degree of homology between the IncF plasmids (**b**). Genetic maps and degree of homology between the resistance gene cassettes of AGR4587, AGR5151 and AGR6128 (**c**). Genes coloured in grey are hypothetical proteins, *bla*_CTX-M-27_ is coloured in red, other resistance genes are coloured in blue and the *qacE* gene is coloured in yellow.

A core gene analysis of the IncF plasmids with their closest relatives (Table S1) was carried out (Fig. 4a). ESBL-producing ST131 strains have previously been found to predominantly carry IncF plasmids with multiple plasmid replicons including the IncFII, IncFIA and/or IncFIB types [68]. IncF-carrying *bla*_CTX-M_ plasmids containing the multiple plasmid replicons IncFII, IncFI and Col156 representing sequence type [F1:A2:B20] have been associated with STs other than ST131, including ST38 [62]. The IncF plasmids from the three ST131 strains contained the same backbone and similar resistance genes but were distinct from the ST69 IncF plasmid (Fig. 4a,b). However, differences arose in their type and number of transposases. For example, in AGR6128 *bla*_CTX-M-27_ is flanked by an IS6 family transposase IS26 on either side, whereas in AGR5151 *bla*_CTX-M-27_ is flanked by an IS6 family transposase IS15 and an IS5 family transposase IS903 (Fig. 4c).

The closest relatives of the pAGR6137a plasmid were IncHI2 plasmids previously isolated from *Enterobacter* spp. (Table S2). This IncHI2 plasmid has been isolated from a variety of *Enterobacteriaceae* species including the *Enterobacter cloacae* complex, *Salmonella enterica *, *Klebsiella pneumoniae *, *Citrobacter freundii* and *E. coli* [78–81]. It has been suggested that the reason for the successful spread of this IncHI2 plasmid is because it harbours numerous metal resistance genes [80]. Studies suggest that the *ter* operon, encoding tellurite resistance, is found on all IncHI2 plasmids [82].

In conclusion, the isolation and whole genome sequencing of the four ESBL-producing *E. coli* isolates collected from a local freshwater site shows the importance of taking a One Health approach in understanding the sources and transmission pathways of AMR bacteria. The use of long-read sequencing enabled the genetic context of the *bla*_CTX-M-27_ and *bla*_CTX-M-15_ genes to be elucidated. Additionally, differences in the order and content of resistance gene cassettes between strains highlight the malleability of resistance genes and their associated mobile elements.

## Supplementary Data

Supplementary material 1
